# Institution within institution

**DOI:** 10.1308/rcsann.2024.0080

**Published:** 2024-09-01

**Authors:** B Rogers

There exist institutions within institutions, and the Technical Section of the *Annals* is an example of such. I would estimate that nearly every surgeon in the UK has at one time read through this section and gleaned a novel technical tip. Furthermore, I would suggest that today, a surgeon somewhere in the world is using a technique described in this section of the journal. It is a unique feature in surgical publishing and highlights the pan-surgical academic breadth of the *Annals* in general. The techniques are frequently practical and simple, facilitating and augmenting existing surgical techniques. Indeed, the section affords the cross-fertilisation of knowledge, whereby surgeons of all specialties benefit.

The success of this section, and the associated surgical benefits, are a reflection upon the dedication, experience and insight of Professor Bruce Campbell, who is stepping down as editor of the Technical Section of the *Annals* after more than 25 years. It is not an overstatement to say that the entire surgical profession owes him a huge thanks. The profession relies upon people doing unpaid work in their own time for the ultimate benefit of colleagues and patients. Bruce is continuing to support surgery, having been elected to leadership roles within the Royal Society of Medicine.

On a personal note, his knowledge and support have been invaluable to me as Editor-in-Chief, and I thank him for this. In addition, every time I do an intramedullary femoral nail, I use techniques I learned through this section!

The *Annals* resides as the academic publication of the Royal College of Surgeons of England, presenting clinical-based surgical research following blinded peer review. This has been the *raison d’être* since its inception in 1947 with its first editor Sir Cecil Wakeley. Some articles bring this more into focus and stimulate debate, which benefits the whole profession. Readers should be assured that all articles published have been single-blind peer-reviewed by colleagues, and as such, the journal is editorially independent. It is important that articles are read and discussed, rather than simply cited, and clearly views may vary on the conclusions made. Being a reviewer for the *Annals* affords an excellent opportunity to develop critical appraisal skills while remaining abreast of advances in surgery. We always welcome further reviewers.

As for all scientific journals, if published research in the *Annals* fosters debate and discussion, the profession will gain through that. Furthermore, because of its editorial independence it is not the remit of the *Annals* to set policy or standards, that being the role of multiple professional bodies - including the Royal College of Surgeons of England.

Lastly, I was delighted to be present at the Diplomates ceremony at the College on 9 July 2024 where Mr Michael Bath was awarded the Sir Cecil Wakeley medal, as the author of the best original research paper by a trainee in 2022.^1^ He received the award from the President of the College, Mr Tim Mitchell. This award, presented annually, is a great achievement considering the number and standard of articles submitted and published. In a small way, this award is an ‘institution within an institution’ as is Prof Bruce Campbell.

It is important we value these, as they form part of the fabric of our profession.

**Figure:**
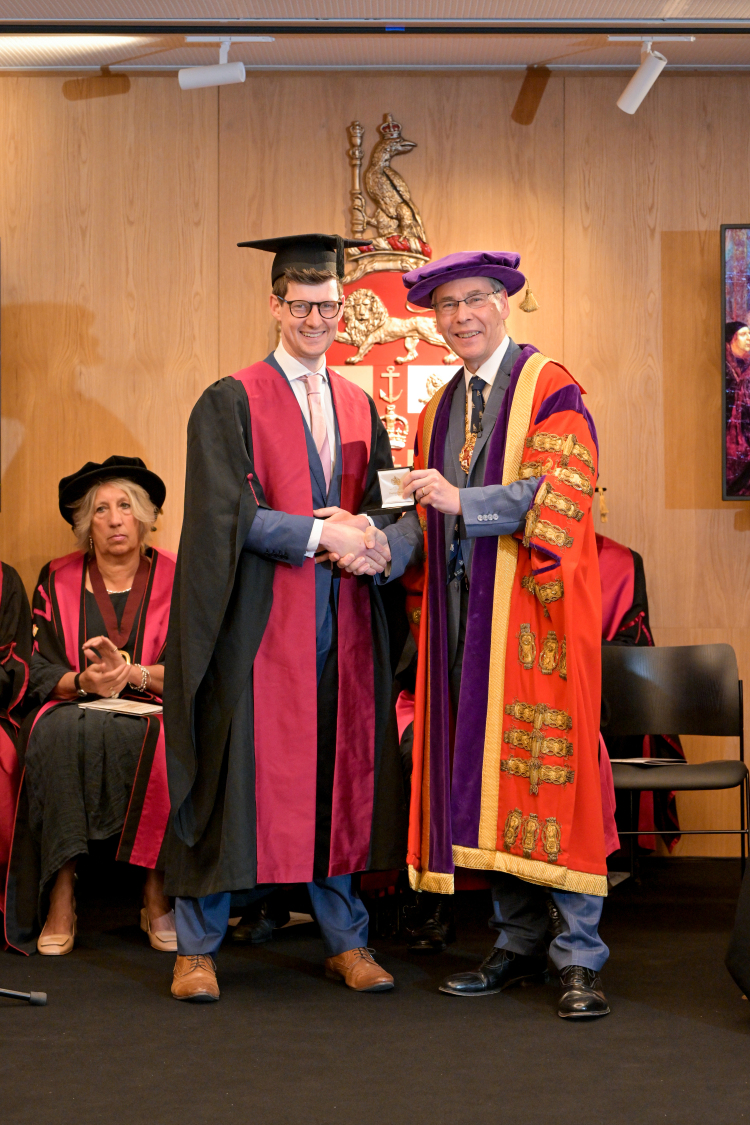
Mr Michael Bath receiving the Sir Cecil Wakeley medal from RCS England President Tim Mitchell
